# How to Be Beautiful

**Published:** 1891-06

**Authors:** 


### HOW TO BE BEAUTIFUL.

Beauty has its foundation in physical well-being, which must be understood and obeyed, these laws being clearly indicated in our physical and mental constitutions. They demand—1. Proper food and drink in such quantities as the system is capable of readily assimilating. 2. Air and sunlight in abundance. 3. Sufficient exercise, rest and sleep. 4. An agreeable temperature. 5. Perfect cleanliness. The whole secret of a full form and rosy cheeks lies in pure blood, manufactured from wholesome food by healthy and active vital organs.

